# Mechanical Behavior of Hybrid Glass/Steel Fiber Reinforced Epoxy Composites

**DOI:** 10.3390/polym9040151

**Published:** 2017-04-23

**Authors:** Amanda K. McBride, Samuel L. Turek, Arash E. Zaghi, Kelly A. Burke

**Affiliations:** 1 Civil and Environmental Engineering Department, University of Connecticut, 261 Glenbrook Road, Unit 3037, Storrs, CT 06269-3037, USA; samuel.turek@uconn.edu; 2 Chemical and Biomolecular Engineering, University of Connecticut, 191 Auditorium Road, Unit 3222, Storrs, CT 06269-3222, USA; 3 Polymer Program, Institute of Materials Science, University of Connecticut, 97 North Eagleville Road, Unit 3136, Storrs, CT 06269-3136, USA; 4 Biomedical Engineering, University of Connecticut, 260 Glenbrook Road, Unit 3247, Storrs, CT 06269-3247, USA

**Keywords:** composite, hybrid, fiber reinforced polymer, mechanical properties, plastic deformation, energy absorption

## Abstract

While conventional fiber-reinforced polymer composites offer high strength and stiffness, they lack ductility and the ability to absorb energy before failure. This work investigates hybrid fiber composites for structural applications comprised of polymer, steel fiber, and glass fibers to address this shortcoming. Varying volume fractions of thin, ductile steel fibers were introduced into glass fiber reinforced epoxy composites. Non-hybrid and hybrid composite specimens were prepared and subjected to monolithic and half-cyclic tensile testing to obtain stress-strain relationships, hysteresis behavior, and insight into failure mechanisms. Open-hole testing was used to assess the vulnerability of the composites to stress concentration. Incorporating steel fibers into glass/epoxy composites offered a significant improvement in energy absorption prior to failure and material re-centering capabilities. It was found that a lower percentage of steel fibers (8.2%) in the hybrid composite outperformed those with higher percentages (15.7% and 22.8%) in terms of energy absorption and re-centering, as the glass reinforcement distributed the plasticity over a larger area. A bilinear hysteresis model was developed to predict cyclic behavior of the hybrid composite.

## 1. Introduction

Fiber-reinforced polymer (FRP) composites are commonly comprised of glass or carbon fibers to provide a high-strength and lightweight material for a variety of industries. However, these fibers are inherently brittle and have a limited energy absorption capacity prior to failure. This prohibits the use of FRPs in certain applications, specifically in critical structural elements that may be subjected to extreme events such as earthquakes, truck collision, or blast, during which energy absorption is crucial. Researchers have studied various ways to improve composite ductility, including polymer matrix toughening via silica nanoparticles [1] and the use of more ductile polymeric (e.g., polypropylene) [2] or natural fibers (e.g., flax) [3]. However, these methods may compromise composite stiffness.

Metal fiber reinforcement has traditionally been used in rubber tires for additional strength in the form of continuous steel cords [4] and in engineered cementitious composites (ECC) for ductility in the form of short fibers [5]. Advancements in the manufacturing of metal fibers have introduced a unique class of ultra-thin (<100 μm) stainless steel fibers, possessing both a high stiffness and failure strain. Recent studies have been performed on the tensile and impact behavior of unidirectional (UD) and cross-ply polymer composites utilizing these steel fibers [6,7,8,9,10,11]. These studies have investigated the effect of brittle and ductile matrices, fiber architecture, and modifying adhesion between fiber and matrix. This research has demonstrated the potential of these fibers to enhance structural performance of composites in terms of failure strain and energy dissipation.

Hybrid fiber composites were developed to provide both strong and ductile reinforcement while reducing material cost and weight. One class of hybrids, known as fiber metal laminates (FML), consist of alternating layers of thin metal sheets and FRP [12]. FML are commonly layered with aluminum and glass (e.g., glass laminate aluminum reinforced epoxy (GLARE)) or aluminum and Kevlar (e.g., aramid fiber reinforced aluminum laminate (ARALL)) and are used as an alternative material for airframe structures with enhanced fatigue resistance [13,14]. GLARE has been commercially implemented in the fuselage of the Airbus A380 [15]. Rubio-González and coworkers studied residual strength of carbon-reinforced aluminum FMLs after fatigue damage through open-hole testing, in which it was observed that fiber laminates were more fatigue tolerant than the base carbon composite [16].

Mechanical properties and predictive models of nonmetal hybrid fiber-reinforced composites have been reviewed by Kretsis, and more recently by Swolfs and coworkers, who also reviewed metal fiber composites [17,18]. The ‘hybrid effect’ was investigated by Hayashi and defined as the apparent failure strain enhancement of the low elongation fiber (carbon) in a carbon/glass hybrid composite [19]. Marom and coworkers defined the hybrid effect as the deviation of mechanical properties from the Rule of Mixtures (ROM) [20], expanding the effect to other properties beyond failure strain. In composites with elastic fibers, longitudinal material properties, such as stiffness and strength, can be estimated based on the Voigt model or “equal strain” assumption of a viscoelastic material in parallel. This leads to the ROM, or weighted mean of various properties [21]. That assumption is no longer valid when incorporating inelastic fibers such as steel; therefore, the prediction of the hybrid composite properties using the ROM becomes less accurate outside the elastic region. More research is needed to understand the mechanical behavior in nonmetal-metal reinforced hybrid composites, however, as the literature is presently limited.

Incorporating metal fibers into conventional composites has previously been studied for their effect on non-structural properties. For example, Breuer and coworkers investigated a carbon/steel fiber hybrid composite as a lightweight material for electrical conductivity in aerospace applications [22]. However, it was realized that composite mechanical properties can also be further tailored using metal fibers for structural benefits. Satish and coworkers studied the tensile and compressive behavior of a steel/nylon fiber reinforced polyester composite [23]. The addition of steel greatly increased the strength and stiffness of the composite, as those properties of steel are superior to nylon and polyester. However, delamination failure was observed due to a weak interface formed between the steel and the polyester. Ahmed studied composite multifunctionality by considering the impact behavior of the hybridization of glass and steel reinforcement, observing that the addition of metal fibers provides increased energy absorption and lowered the damage area under low velocity impact [24]. Thysen studied the effect of lay-up and fiber ratios on the tensile strength and failure strain of glass/steel composites in epoxy and nylon (PA-6) matrices [25]. Without reinforcement, the nylon matrix was more ductile than the epoxy matrix. However, the failure strain of the reinforced epoxy composite displayed higher failure strains than the reinforced nylon composite. These results guided the matrix choice for the experimental study presented in this paper. It was concluded that the hybrid effect was more prominent with steel on the outside and glass on the inside due to differences in thermal expansion inducing an initial compressive stress on the glass fibers after manufacturing. Differences in lay-up were only observed after failure of the glass fibers.

This paper presents the results of an experimental investigation on the mechanical properties, energy dissipation capacity, and strain re-centering ability of composites comprised of glass and steel fibers in an epoxy matrix. Within the hybrid composites, the effect of the glass-to-steel fiber volume ratio was studied. Coupons with and without holes were tested under monolithic and half-cyclic tensile loading to obtain stress-strain relationships, hysteresis behavior, and failure mechanisms. The validity of the ROM using the ‘equal strain’ assumption was studied for the hybrid specimens. Failure specimens were examined to characterize the damage progression due to the interaction of glass and steel fibers. A bilinear hysteresis model was used to confirm the hybrid composite cyclic behavior. The behavior after damage, energy dissipation during loading, and re-centering capabilities of the different hybrids are of interest to ascertain the applicability of hybrid composites in structural engineering.

## 2. Materials and Methods

### 2.1. Research Methodology

An experimental study was conducted to investigate different compositions of a hybrid composite comprised of glass and steel reinforcing fibers. Non-hybrid composites were prepared as baseline comparisons for each fiber type. The composites included glass, steel, and three hybrids with different glass-to-steel fiber ratios. As overall fiber volume fractions were kept approximately constant, glass-to-steel ratios were varied to investigate the differences in mechanical performance and failure mechanisms of the different compositions. Table 1 summarizes the compositions that were prepared and tested. The layer notation is as follows: G = glass fiber and S = steel fiber. As all fibers were oriented in the 0° direction (unidirectional), the fiber direction is omitted from the layer notation; this was also the direction of all loading. In the non-hybrid composites, repeated layers are shown using subscripts. For example, [G]_5_ represents 5 layers of unidirectional (UD) glass fiber fabric and [SGSGSGS] represents alternating layers of steel and glass. The hybrids (Table 1) were designed to have anticipated glass-to-steel ratios of 70/30, [SGGGGS], 50/50, [SGSGSGS], and 30/70, [SSSGSSS]. The calculated fiber fractions are presented in Section 2.3.

Monolithic and half-cyclic tensile testing was conducted. The monolithic tensile testing provided the load-displacement data to find the ultimate tensile strength, stiffness, and failure strain. Open-hole testing is the standard mechanical testing for polymer matrix composites and was performed to observe the effect of a stress concentration on the mechanical properties of the composites. Open-hole testing also helped dictate failure within the gauge length. The results from the open-hole half-cyclic testing were used to find the energy dissipated and residual strain throughout damage cycles. This was used to characterize the strength, energy absorption, and re-centering capabilities of this hybrid material towards exploring the potential for use as structural composites.

### 2.2. Raw Materials

#### 2.2.1. Reinforcing Fibers

**Figure 1:**
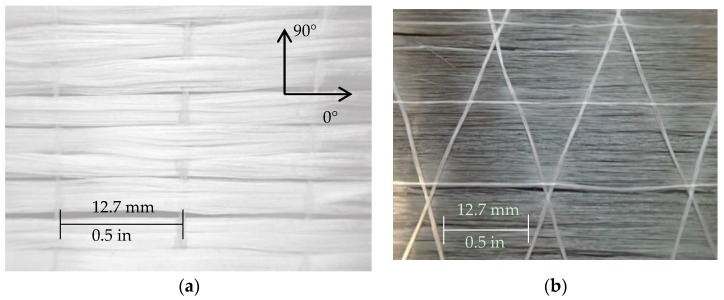
Reinforcement types: (**a**) UD glass fibers; (**b**) UD steel fibers.

Figure 1 shows the reinforcement, glass and steel fibers, investigated in this study. Table 2 presents the properties of these fibers, as provided by the manufacturer The glass reinforcement is a quasi-UD woven roving consisting of Advantex^®^ E-CR glass fibers in the warp (0°) direction provided by Owens Corning (Toledo, OH, USA) (Figure 1a). The fibers in the weft (90°) direction are only present to bind the fiber bundles together and do not contribute to the mechanical properties. Advantex^®^ is a boron-free, corrosion resistant material that is recommended for use in acidic environments.

The steel reinforcement studied is a quasi-UD weave consisting of Grade 316 stainless steel fibers (Figure 1b) in the warp direction and polyethylene succinate (PES) cross yarns, provided by NV Bekaert SA (Deerlijk, Belgium) [26]. The PES yarns serve to control the spacing between the steel fibers and maintain the integrity of the weave; the contribution to the mechanical properties of the fabric is negligible. They were manufactured using a bundle drawing technique [27] and annealed at > 800°C (1472° F) to ensure high strain-to-failure without compromising stiffness.

#### 2.2.2. Matrix

The two-part thermosetting matrix system used for these studies is EPON 828, a difunctional bisphenol A/epichlorohydrin derived liquid epoxy resin, with EPIKURE 3055, an aliphatic amine hardener, as a curing agent supplied by Hexion (Columbus, OH, USA). It was mixed with a manufacturer-recommended resin-to-hardener weight ratio of 2:1 to obtain optimal polymer cross-linking. EPON 828 has become a widely used industry resin because of its mechanical versatility and high resistance to a broad range of chemicals [28]. EPIKURE 3055 hardener has a low viscosity with extended pot life, which improves the workability of the matrix. This allows for a faster impregnation of the fibers. For structural applications, thermosets are preferred over thermoplastic resins for superior creep resistance over a wider range of temperatures. For comparison, monolithic tensile testing per American Society for Testing and Materials (ASTM) D638 [29] was performed on the epoxy (no composite fillers) to characterize the stress-strain properties (elastic modulus (*E*), ultimate tensile strength (*σ*_ult_) and failure strain (*ε*_ult_)) of the matrix (*E* = 2.56 GPa, *σ*_ult_ = 56.9 MPa, *ε*_ult_ = 5.06%).

### 2.3. Manufacturing of Composite Specimens

**Figure 2:**
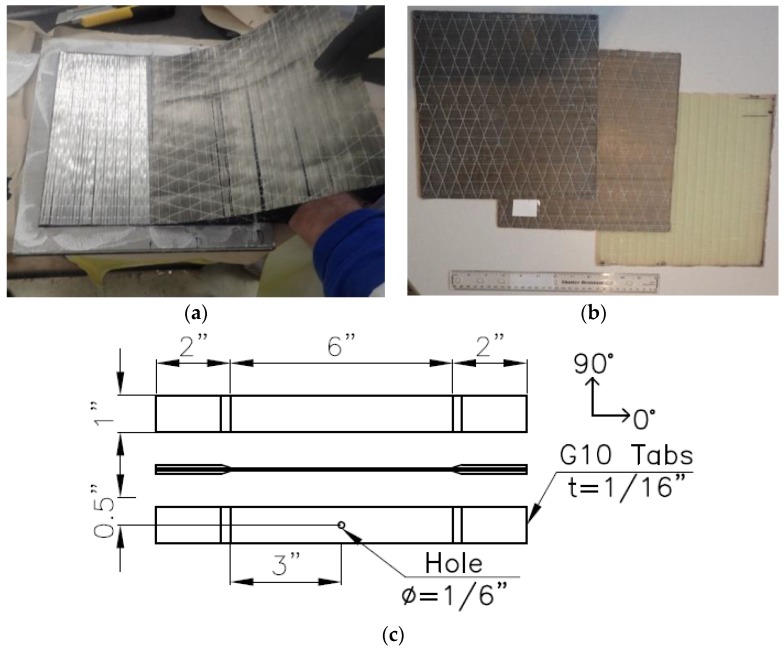
Composite preparation: (**a**) Fiber hand lay-up; (**b**) cured composite plates; (**c)** coupon dimensions with end tabs.

The composite specimens were manufactured using a hand lay-up technique. The reinforcement was oriented on a 25.4 cm × 25.4 cm (10 in × 10 in) square steel plate in a 1.8 mm (0.07 in) thick frame and saturated with epoxy (Figure 2a). The composite plates were cured using the compression molding method. Curing was executed at 93 °C (200 °F) for 2 h as per the manufacturer’s recommendation and under a pressure of 7 bar (100 psi) to allow excess resin to bleed out while reaching the desired thickness. The plates (Figure 2b) were then cooled for approximately 30 min under atmospheric pressure. Care was taken to minimize voids throughout the manufacturing process. After curing, the plates were cut into coupons with dimensions as shown in Figure 2c (254 mm length × 25.4 mm width × 1.78 mm thickness). G10 fiberglass beveled end tabs were applied using Loctite 4014 instant adhesive (Henkel Adhesives, Westlake, OH, USA). The end tabs were necessary to avoid premature failure due to stress concentrations at the testing grips. For a number of each of the specimens specified in Section 2.1, a hole was drilled in the center with a diameter equal to 1/6 of the coupon width, per ASTM specifications. Prior to testing, specimens were inspected to ensure there was no damage surrounding the hole.

The layup compositions of the hybrids were selected to ensure all composites were symmetric so as not to introduce any bending-extension coupling. However, the effect of fiber layup was not explicitly studied. The composites were designed so that the total fiber volume fractions were comparable. Table 3 presents a summary of the actual fiber volume fractions of the composites manufactured in this study. The volume fractions (*v*_f_) were determined using Equation (1) [21], based on the composite thickness (*t*), fiber density (ρ), and fabric areal density (*A*_w_), and number of fiber layers (*n*) using the following equation:

*v*_f_ = (*n* × *A*_w_)/(ρ × *t*). (1)

### 2.4. Experimental Methodology

Composite coupons were first tested under monolithic tensile loading to obtain the ultimate tensile strength (*σ*_ult_) and failure strain (*ε*_ult_) of different material compositions. The testing was conducted on a hydraulic MTS 810 test machine (Eden Prairie, MN, USA) and performed according to ASTM D3039 [30]. The displacement was applied at 2 mm/min (0.08 in/min), the load was recorded using an 89 kN (20 kip) load cell, and the longitudinal strain was measured using a strain-gauge based extensometer with a 10.16 cm (4 in) gauge length and a 350 Ω resistance. All data output was collected via an HBM data acquisition system. All tests were conducted under standard laboratory conditions at room temperature. Four coupons of each composite type were tested under monolithic tension and failure inside the gauge length, away from the grips, was successfully achieved for all specimens.

To investigate energy dissipation and re-centering capabilities of this hybrid material, monolithic (2 coupons) and half-cyclic tensile (2 coupons) tests were then performed on open-hole specimens for each composite type. The open hole simulates realistic stress concentrations that can be introduced in the material and may arise from bolt holes for structural connections or general accumulated damage, for example. First, monolithic open-hole tensile (OHT) testing was performed on each coupon according to ASTM D5766 [31] to obtain the open-hole ultimate tensile strength. Per the standard, this is calculated using the gross cross-sectional area, disregarding the presence of the hole. The true strength (*σ*_true_) was calculated adjusting for reduced area. Next, open-hole half-cyclic (OHC) tensile testing was performed. The loading protocol began and returned to a benchmark of 445 N (100 lbs) as the applied load was increased by 0.1 *σ*_ult_ for each cycle at a constant load rate of 111 N/s (25 lb/s) until failure.

## 3. Results and Discussion

All experimental results are presented in Table 4. For monolithic testing, average values are reported with standard deviation. For open-hole tension and cyclic testing, average values are reported.

### 3.1. No-hole Composites

#### 3.1.1. Tensile Properties

**Figure 3:**
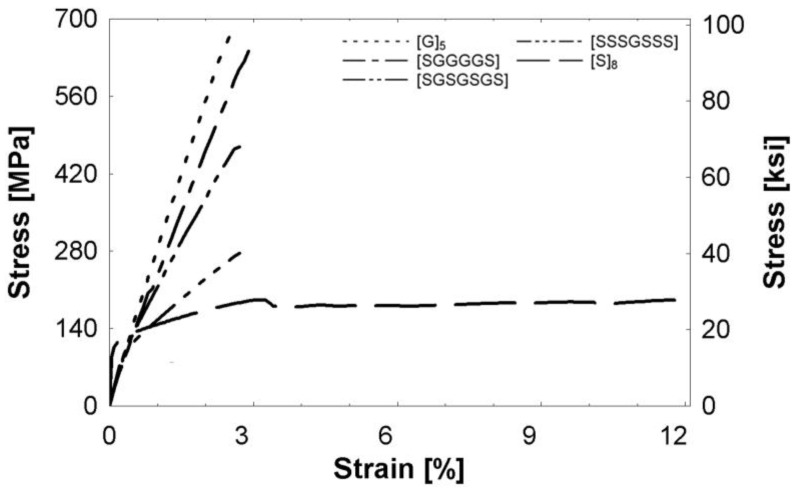
Monolithic tensile stress-strain relationships of specimens with no holes.

The stress-strain behavior of all composites is presented in Figure 3. Following the elastic limit, all composites continue carrying increasing stresses until the ultimate stress is reached. The unidirectional (UD) glass composite, [G]_5_, displayed the largest ultimate strength at 667 MPa (96.7 ksi) and the lowest failure strain of 2.50%. The UD steel fiber composite, [S]_8_, had a significantly larger failure strain of 12.0%, but the lowest ultimate strength. The tensile tests showed a clear nonlinear response for the hybrid composites. The [SGGGGS] hybrid had a strength of 642 MPa (93.1 ksi). As the amount of glass decreased, the ultimate strength also decreased. The [SGSGSGS] and [SSSGSSS] specimens had ultimate strengths of 469 MPa (68.0 ksi) and 276 MPa (40.0 ksi), respectively. The hybrid failure strains were 2.90%, 2.71%, and 2.71%, in order of increasing steel fiber percentage, and did not show a significant difference between the hybrid compositions. These results suggest that adding steel increased the failure strain of glass/epoxy composites. The failure patterns are discussed in Section 3.1.2 to obtain a greater understanding of the stress-strain behavior.

**Figure 4:**
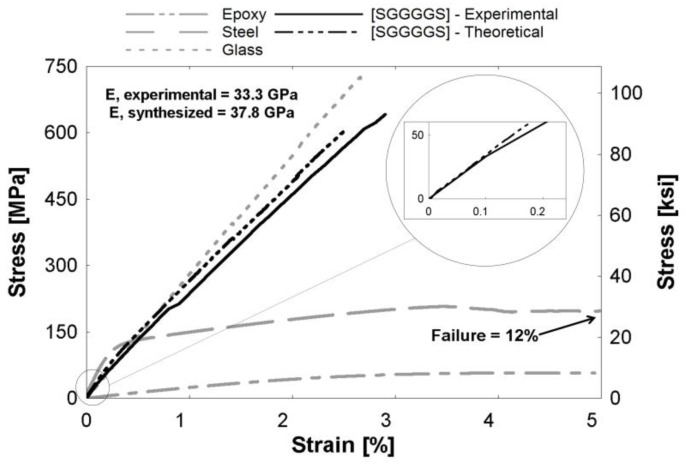
[SGGGGS] hybrid composite curve. (Inset) The linear region expanded from the encircled region.

**Figure 5:**
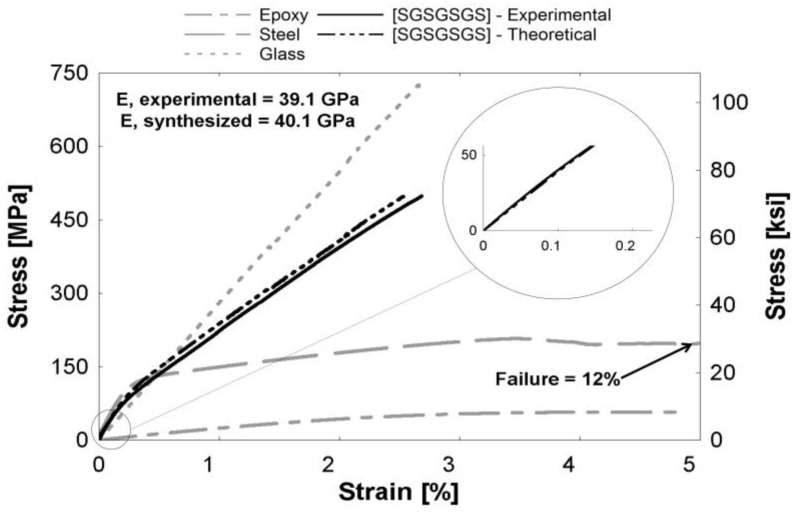
[SGSGSGS] hybrid composite curve. (Inset) The linear region expanded from the encircled region.

**Figure 6:**
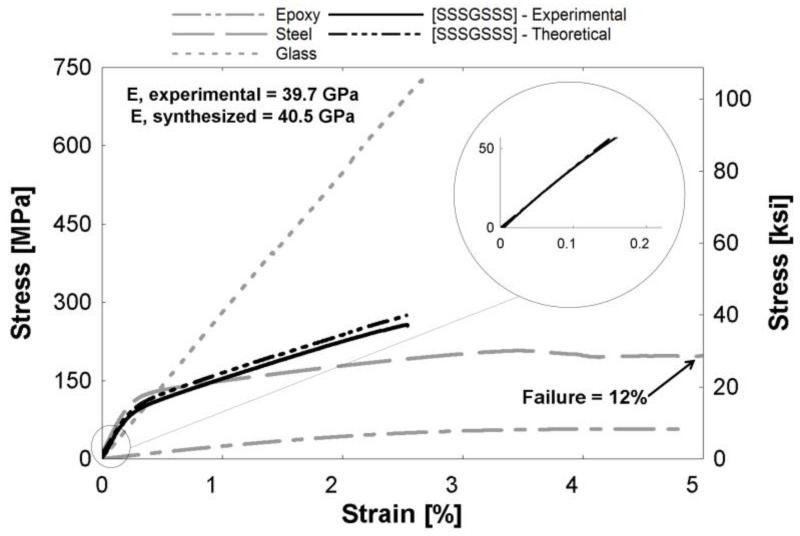
[SSSGSSS] hybrid composite curve. (Inset) The linear region expanded from the encircled region.

Figure 4, Figure 5 and Figure 6 depict theoretical tensile stress-strain relationships of curves calculated from theory using the rule of mixtures (ROM), which sums the stresses of individual constituents based on volume fraction of the hybrid composites. The figure also presents the actual experimental curve. The glass and steel curves (Figure 4, Figure 5 and Figure 6) were obtained by subtracting the contribution of the epoxy matrix, thus the epoxy matrix is shown as its own entity. These figures show that the ROM was valid in the elastic region, as seen by the prediction of the stiffness in the linear region (Figure 4, Figure 5 and Figure 6 insets). Slight differences between curves can be attributed to limitations in the accuracy of the volume fraction measurement or minor defects, such as fiber misorientation and resin voids. Both Callens [6] and Thysen [25] discuss a lower experimental stiffness than theoretically predicted due to fiber misorientation in the manufacturing process. After the steel begins to yield at approximately 0.2%, the rule of mixtures slightly over-predicts the stress-strain relationship. The experimental curve is consistently 92%–95% of the theoretical curve in the plastic region. Additionally, the failure strain of the experimental hybrid is consistently greater than the theoretical (as calculated from the ROM) value for all hybrids. This confirmed that there was a synergistic effect on failure strain due to fiber hybridization.

#### 3.1.2. Failure Mechanisms

**Figure 7:**
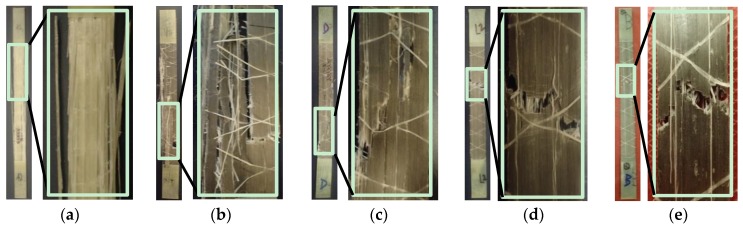
Images of failure specimens: (**a**) [G]_5_; (**b**) [SGGGGS]; (**c**) [SGSGSGS]; (**d**) [SSSGSSS]; (**e**) [S]_8_.

**Figure 8:**
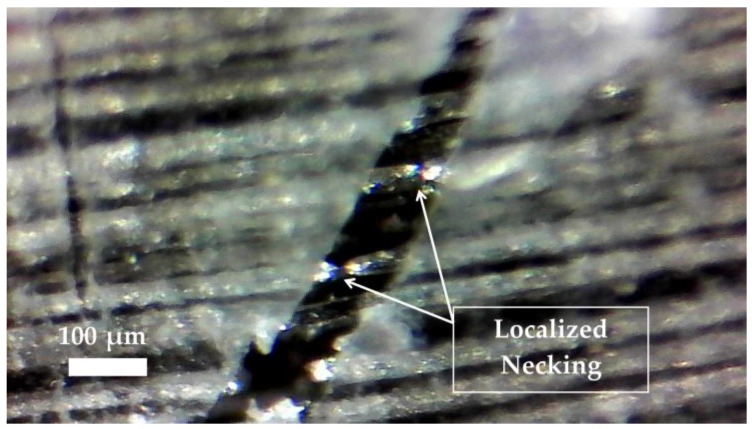
UD steel composite crack bridging and necking at matrix crack. Image taken by Carson digital handheld microscope.

The failure of specimens was visualized using a digital camera (Olympus Stylus Tough TG-4 16.0 MP Compact from Olympus America (Center Valley, PA, USA)) to characterize the response of the composite specimens to the monolithic stress-strain testing. For each composite tested, all samples displayed similar and reproducible failure patterns. The general failure mechanism of the UD glass composites, [G]_5_, can be described as a “brooming” effect as described by Harik et al. [32], and shown in Figure 7a. This is a result of scattered fiber breakage and debonding from the matrix. As shown in the stress-strain plots (Figure 3), the failure was sudden and brittle. This random brooming effect confirms the stochastic failure and high energy release of the fibers at multiple locations. The UD steel fiber composites, [S]_8_, exhibited failure normal to the direction of loading, as shown in Figure 7e. The fracture cross-section was not in-plane, indicating there was fiber pullout and necking, also observed by Callens et al. [6]. At the presence of a matrix crack, the fibers start to yield and therefore produce a more localized failure, visible in the microscope image of Figure 8 (Carson digital handheld microscope from Carson Optical Inc. (Ronkonkoma, NY, USA)). The steel fibers maintained the integrity of the composite after the matrix cracks started forming. This is also evident as the composite continues to yield beyond the failure strain of the epoxy. This composite showed high ductility, and the failure strain of 12.0% was more than twice that of pure epoxy (5.06%).

The three different hybrid composite failure patterns were observed (Figure 7b–d), and these patterns lay within a spectrum depending on the respective fiber fractions. Hybrids with higher percentages of glass exhibited similar failures to [G]_5_, where failure was distributed along the length. Assuming the glass fibers fail first in a random pattern, the stress was transferred to the neighboring steel fibers at many different locations along the gauge length. It is clear that the failure is distributed more axially. This resulted in larger failure strains than the [G]_5_ specimen (Figure 3), and a spread of plasticity along the length of the specimen. In contrast, hybrids with higher percentages of steel resembled the localized [S]_8_ failure more closely. Although the failure strain of the composites was not nearly as high as the [S]_8_ composite (Figure 3), these results suggest that the presence of steel yielding can afford warning to potential structural failure. An identical failure spectrum was observed in both the open-hole tensile and open-hole cyclic specimens.

### 3.2. Open-Hole Composites

#### 3.2.1. Tensile Properties

**Figure 9:**
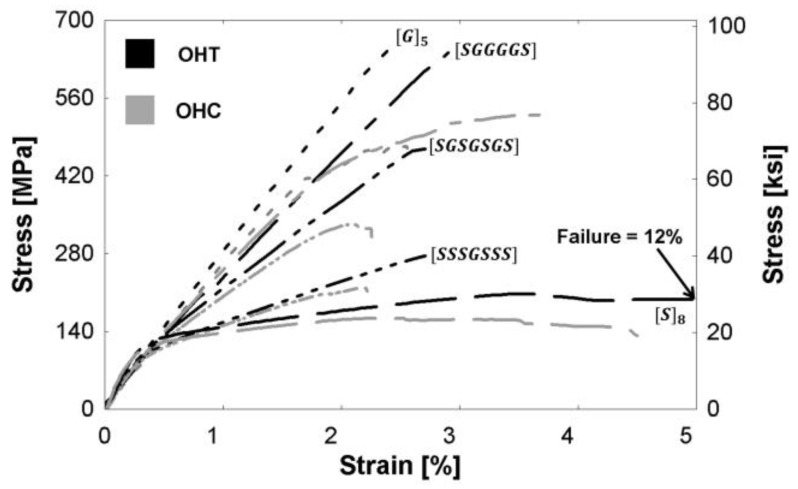
Tensile stress-strain relationships of specimens without holes and with holes (denoted by the label “OHT”).

Figure 9 presents a comparison of the monolithic tensile stress-strain relationships of composites with and without a hole. The ultimate nominal tensile strength (*σ*_ult_), failure strain (*ε*_ult_), and true tensile strength (*σ*_true_) are reported for each specimen in Table 4. The stiffness of the open-hole (OHT) specimens remains unchanged compared to specimens without a hole, but there is a decrease in the ultimate tensile strength of the OHT specimens due to the reduction in the specimen’s cross-sectional area. The true stress of the OHT specimen was calculated using the reduced area of the specimen, and the ratio of the true stress to the ultimate tensile stress of the specimen without a hole, $\frac{\sigma_{\mathrm{true}}}{\sigma_{\mathrm{ult},\mathrm{no}\;\mathrm{hole}}}$, is presented in Table 4. Though the OHT specimens were fewer in number and hence no statistical testing was possible for these specimens, qualitatively it can be noted that the presence of steel decreased the susceptibility of the composites to stress concentrations, as indicated by ratios closer to unity. Hybrid (steel with glass) epoxy composites are thus better able to retain more of the composite’s ultimate strength even with a stress concentration present in contrast to the more conventional glass/epoxy FRP composite.

**Figure 10:**
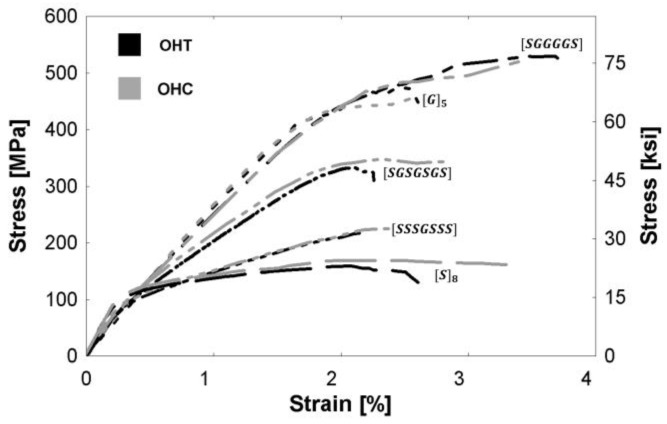
Stress-strain relationships of specimens with open-holes during monolithic tensile (denoted OHT) and half-cyclic loading (denoted OHC).

Figure 10 presents open-hole specimen stress-strain relationships. The open-hole tensile curves (OHT) and the backbone of the open-hole half-cyclic curves (OHC) are shown for all compositions. The ultimate strength and failure strains are presented in Table 4. The OHT tests were displacement-controlled, and the OHC tests were load-controlled. No significant differences were observed between the stress-strain behavior during tensile and half-cyclic loading. This suggests that the accumulated damage from loading/unloading below the ultimate stress does not have a significant effect on the composite’s mechanical properties.

#### 3.2.2. Cyclic Properties

**Figure 11:**
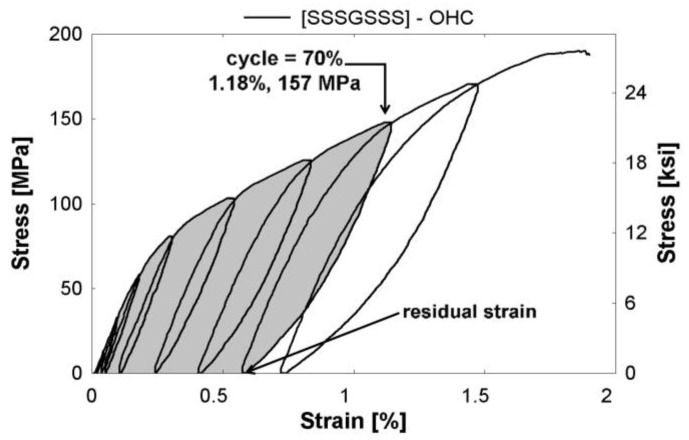
Area under the curve of half-cyclic loading for [SSSGSSS]. The seventh loading cycle is demonstrated by an arrow.

Figure 11 shows a sample of stress-strain behavior after half-cyclic tensile loading of an open-hole specimen, [SSSGSSS]. In this testing, the loading protocol began and returned to a benchmark of 445 N (100 lbs) as the applied load was increased by 0.1 *σ*_ult_ for each cycle at a constant load rate of 111 N/s (25 lb/s) until failure. For example, the maximum stress and strain of the seventh cycle, where the sample was loaded to a stress equal to 0.7 *σ*_ult_, is indicated on the figure to demonstrate how energy dissipation was found at the end of each loading cycle. The area under the curve is shaded to signify the amount of energy absorbed in each loading cycle. Residual strain caused from plastic deformation was assumed to be the strain at the end of each unloading cycle.

**Figure 12:**
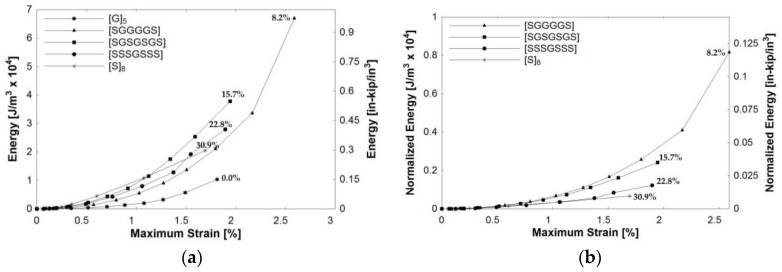
Energy dissipation of composites during open-hole half-cyclic loading: (**a**) calculated energy values; (**b**) normalized energy values per 1% of steel. (Note) Percentage along curves represents the steel fiber fraction in the composite. Lines connecting points used to display trend.

Figure 12a plots the amount of energy dissipated during open-hole half-cyclic loading of the composites. The x-axis represents the maximum strain reached at each cycle prior to unloading. The y-axis represents the energy dissipated during each loading cycle found by integrating the stress-strain curve. The percentage adjacent to each curve represents the total volume fraction of steel within the composite. For example, for a perfectly elastic material with no permanent deformation, the energy dissipated will be near zero prior to failure, as the material unloads along the same linear path. This was observed in specimen [G]_5_. In contrast, plastic materials will dissipate energy due to yielding during loading. In a structural application, this could potentially lead to warning of damage prior to failure, which is ideal in structural design. High failure strains of UD steel composites lead Callens et al. [7] to study energy dissipation of UD steel composites through impact testing.

A composite comprised of high strength elastic glass fibers and ductile steel fibers was expected to have a large area under the stress-strain curve, due to a high amount of energy dissipated. The [SGGGGS] hybrid, consisting of only 8.2% steel, was found to dissipate the most energy. Since the strength of the glass is significantly higher than the steel fibers, this hybrid does not need a large amount of steel to achieve high energy dissipation. To facilitate comparison among composite compositions with differing amounts of glass, the normalized energy value per 1% of steel present in the composite is plotted versus maximum strain in Figure 12b. The results of Figure 12a,b are consistent: hybrids containing less steel were able to dissipate more energy.

**Figure 13:**
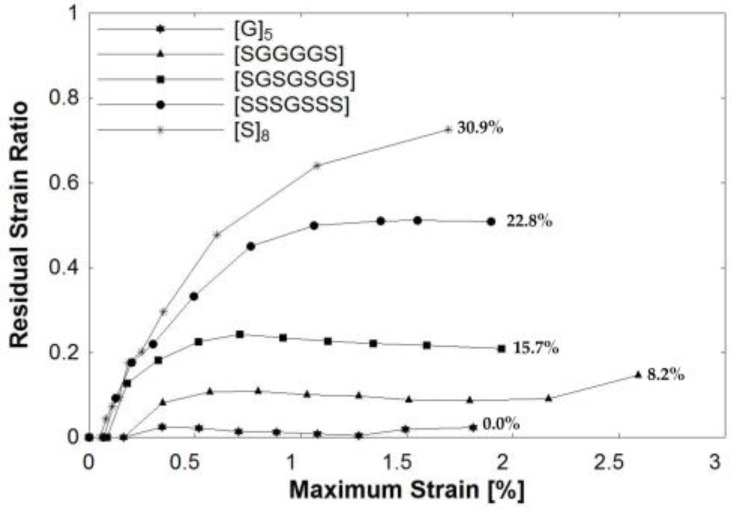
Residual strain ratio of composites during open-hole half-cyclic loading. Note: Percentage along curves represents the steel fiber fraction in the composite. The lines connecting points are used to display trends.

The main goal of the nonmetal-metal hybrid composite system is to improve ductility and energy absorption prior to failure. However, ductility causes permanent deformation and residual strain over the material lifecycle. A material that has re-centering capabilities after loading and unloading is appealing for structural applications for both strength and serviceability limit states. Figure 13 shows the residual strain behavior after open-hole half-cyclic loading. The x-axis represents the maximum strain reached at each cycle prior to unloading. The y-axis represents the residual strain ratio, which is calculated as the residual strain at the end of a loading cycle divided by the cycle’s maximum strain. The closer the ratio is to zero, the better the re-centering capability of the material. For a perfectly elastic material with no plastic deformation, the residual strain ratio will be near zero. This behavior was observed in the [G]_5_ specimen. In contrast, an inelastic material has a ratio closer to one, as observed in the [S]_8_ specimen. Of the three hybrid composites, the composite with the lowest percentage of steel, [SGGGGS], had the lowest residual strain ratio, plateauing around 0.12. This signifies very good re-centering capabilities. The other hybrids also had more favorable re-centering capabilities with relatively low ratios that plateaued after reaching a threshold value for the strain ratio. A material that has a low and consistent residual strain ratio along loading cycles indicates greater stability. Structural design relies on parameters, such as stability, that characterize the material strength and dictate the response to loading.

#### 3.2.3. Theoretical Hysteresis Model

**Figure 14:**
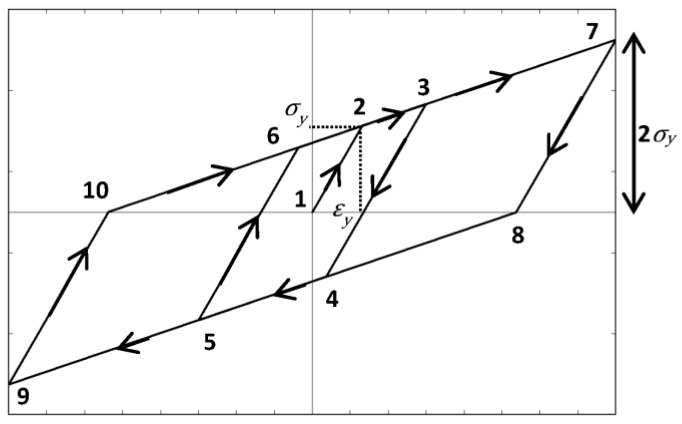
Lead-rubber bearing ideal bilinear hysteresis behavior.

A theoretical model was used to further analyze the hybrid composite cyclic behavior. The combination of an elastic and plastic material results in unique hysteresis behavior. Under half-cyclic testing, it was observed that the hybrid composites resemble the behavior of lead-rubber bearings (LRBs), commonly used as base isolators in structural seismic applications [33]. The novelty in this system lies in the re-centering force. This hybrid composite behavior may be predicted using the bilinear hysteresis model shown in Figure 14. Point 1 begins at (0, 0) and the *x*-value of Point 2 is located at the yield strain of the steel fibers, ε*_y_*. The slope of line 1–2, or the elastic slope, represents the modulus of the hybrid composite in the elastic region. The slope of line 2–3, or the hardening slope, is equivalent to the modulus of the glass and epoxy constituents, as well as the post-yielding slope of the steel. The post-yielding slope was determined by bilinearization of the steel stress-strain relationship using equal areas. This hardening slope is what dictates the return path at the unloading of each cycle. The *x*-value of point 3 represents the maximum strain attained at that respective cycle. The slopes of line 3–4 and line 5–6 are equal to that of line 1–2 and the vertical distance is equivalent to 2 *σ*_y_. Point 7 represents a targeted design strain. The 7–8–9–10 parallelogram signifies the outermost boundary of the ideal bilinear hysteresis behavior.

**Figure 15:**
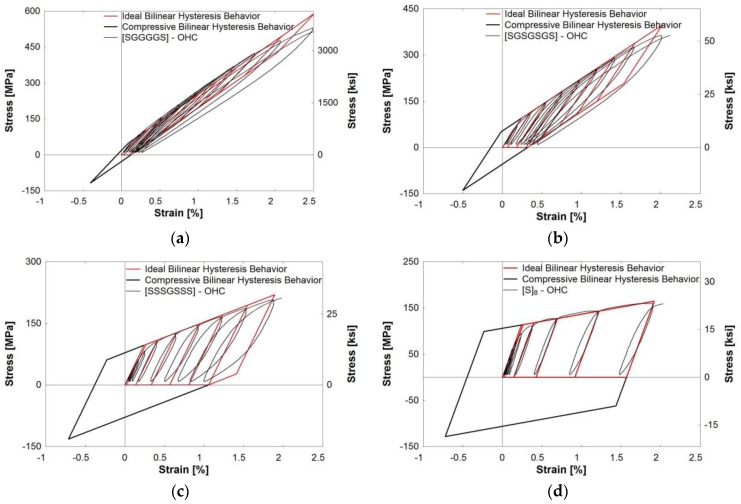
Ideal hysteresis behavior: (**a**) [SGGGGS]; (**b**) [SGSGSGS]; (**c**) [SSSGSSS]; (**d**) [S]_8_.

This ideal hysteresis model was applied to the hybrid composites, [SGGGGS], [SGSGSGS], and [SSSGSSS], along with the steel composite, [S]_8_ (Figure 15). In this work, only half-cyclic testing was performed due to experimental limitations that did not permit the acquisition of compression data. However, the stress-strain curve does appear to follow kinematic hardening behavior. Using this assumption, the complete hysteresis behavior, including stress-strain relationships in the compressive stress region, was predicted and shown in Figure 15. The red portion of the bilinear model followed each experimental tensile loading and unloading cycle. The black portion of the curve serves as the predicted extension for full-cycle behavior by extending the curve into the compression region. This bilinear model predicted the hysteresis behavior of all hybrid composites by finding the elastic and hardening slopes, the yield stress, and the residual strain. Composites with a higher fraction of steel were found to follow the prediction more closely. The calculated elastic and hardening slopes align with the paths of the experimental hysteresis behavior. Hybrid composite cycles that lie within the ideal parallelogram suggest material stability. Once the stress reaches greater than 2 *σ*_y_, the steel fibers may yield in compression. This is not desirable for structural materials because subsequent loading cycles will also yield, and the material will become more unstable over time. The area under each idealized loading cycle was found, and the energy dissipation followed the same trend as the experimental values. As this model is an idealized shape, it can be seen how it can under- or over-predict the actual energy dissipation of the composite depending on the curve fit.

## 4. Conclusions

The conclusions presented herein are based on the results of monolithic tensile and half-cyclic testing of glass/steel fiber reinforced epoxy composite coupons. Three different fiber ratios of steel reinforcement were studied: (1) [SGGGGS] (8.2% steel); (2) [SGSGSGS] (15.7% steel); and (3) [SSSGSSS] (22.8% steel). The mechanical properties were characterized and gave rise to the following findings:

- The tensile strength of the hybrid composites was directly proportional to the respective glass and steel fiber percentages. The strengths from highest to lowest were as follows: [G]_5_, [SGGGGS], [SGSGSGS], [SSSGSSS], and [S]_8_. This order held true for tensile, open-hole tensile, and open-hole half-cyclic loading.
- The rule of mixtures proved valid in the elastic region and predicted the stiffness values accurately. However, in the post-yield region, the ROM consistently over-predicted the stress-strain relationship. More research is needed on theoretical models of nonmetal-metal hybrid composites in the inelastic region.
- Composites with a higher percentage of steel had localized failure. In contrast, composites with higher percentages of glass had a more distributed failure pattern, making it difficult to predict failure location. The addition of steel helped maintain the integrity of the composite after the failure of the glass fibers. The hybrid composites experienced a ductile failure, which may provide warning to structural failure. This was due to the spread of plasticity over a larger length of steel fibers.
- The addition of steel fibers to glass/epoxy composites decreased the vulnerability to stress concentrations. Accumulated damage from cyclic loading does not have a significant effect on the composite stress-strain relationship. This behavior is potentially beneficial in structural elements that are subject to repeated dynamic loading.
- [SGGGGS] outperformed [SGSGSGS] and [SSSGSSS] and offers balanced mechanical properties. This composite had the highest strength, dissipated the most energy during loading, and showed the most consistent re-centering capabilities. This may signify that the amount of steel reinforcement may be optimized to achieve target structural performances.
- The hybrid composite half-cyclic behavior may successfully be predicted using the bilinear hysteresis model of lead-rubber bearings. This model suggested that the hybrid composites had greater stability. This model also justified the experimental energy dissipation and residual strain ratio results.
- Overall, glass/steel fiber hybrid composites show promise in structural applications because of their high strength, energy absorption during loading, and re-centering capabilities. More research is needed to optimize the composite design to achieve higher failure strains.

## Figures and Tables

**Table 1:** Composition of prepared composites.

Composite Type	Layer Notation	Anticipated Fiber Ratio	
		Glass	Steel
UD Glass	[G]_5_	100	0
Hybrid 1	[SGGGGS]	70	30
Hybrid 2	[SGSGSGS]	50	50
Hybrid 3	[SSSGSSS]	30	70
UD Steel	[S]_8_	0	100

**Table 2:** Properties of reinforcing fibers.

Reinforcing Fiber	Aerial Density g/m^2^ (oz/yd^2^)	Fiber Diameter μm (in)	Fiber Density g/cm^3^	Young’s Modulus GPa
UD Glass	327 (9.6)	10 (0.0004)	2.62	82
UD Steel	570 (16.8)	30 (0.0012)	N.S.	193

N.S. = not specified by manufacturer.

**Table 3:** Prepared composite fiber volume fractions.

Layer Notation	Glass Fiber Fraction	Steel Fiber Fraction	Total Fiber Volume Fraction
[G]_5_	34.7 ± 0.1%	-	34.7 ± 0.1%
[SGGGGS]	28.2 ± 0.1%	8.2%	36.4 ± 0.1%
[SGSGSGS]	20.3 ± 0.2%	15.7 ± 0.2%	36.0 ± 0.4%
[SSSGSSS]	6.5%	22.8 ± 0.1%	29.3 ± 0.2%
[S]_8_	-	30.9 ± 0.5%	30.9 ± 0.5%

**Table 4:** Ultimate tensile strength, failure strain, and true strength results of no-hole and open-hole tensile tests.

Composite	No-Hole Tension		Open-Hole Tension				Open-Hole Cyclic	
	*σ*_ult_, MPa (ksi)	*ε*_ult_, %	*σ*_ult_, MPa (ksi)	*ε*_ult_, %	*σ*_true_^1^, MPa (ksi)	*σ*_true_/ *σ*_ult,no-hole_	σ*_ult_*, MPa (ksi)	*ε*_ult_, %
[G]_5_	667 ± 18.4	2.50 ± 0.12%	474	2.46%	569	0.852	455	2.55%
	(96.7 ± 2.67)		(68.7)		(82.5)		(66.0)	
[SGGGGS]	642 ± 35.5	2.90 ± 0.30%	530	3.68%	598	0.932	521	3.41%
	(93.1 ± 5.15)		(76.9)		(86.7)		(75.6)	
[SGSGSGS]	469 ± 29.7	2.71 ± 0.13%	333	2.25%	422	0.900	348	2.83%
	(68.0 ± 4.31)		(48.3)		(61.2)		(50.5)	
[SSSGSSS]	276 ± 11.2	2.71 ± 0.12%	218	2.25%	260	0.941	225	2.34%
	(40.0 ± 1.62)		(31.6)		(37.7)		(32.6)	
[S]_8_	208 ± 6.67	12.0 ± 0.01%	161	4.50%	194	0.932	169	3.32%
	(30.2 ± 0.97)		(23.4)		(28.1)		(24.5)	

^1^ True strength was calculated adjusting for the reduced cross-sectional area due to the hole.
